# The Teeth

**Published:** 1875-07

**Authors:** 


### THE TEETH.

In the face of the universal esteem in which these wonderful structures are held, we find the most widespread carelessness as to their preservation. It is not too much to say that good teeth in adult mouths are the rare exception; they should be the rule. Why are they not the rule? Simply because of the criminal neglect of their owners. What would be said of the man or woman who would rest content to plunge the hands into a mass of meat and grease, and potatoes, and bread and coffee, three times each day, and yet leave the hands unwashed? Yet this is precisely what is done, with reference to the teeth, by ninety-nine one hundredths of the American people. The result is, a breath bearing the pestilence; a thousand diseases induced by breathing air thus contaminated; the early loss of the most beautiful ornaments of the face; dyspepsia from inperfect mastication; the evidences of old age before the years of youth are past; deformity and ugliness where beauty might have dwelt had ordinary neatness been the rule.

The teeth can be saved, ordinarily, by proper care, begun in infancy and never suspended. A brush must be used, and a very substantial one, which should be often renewed. The teeth should be brushed *up and down*, so that the bristles will pass between them and dislodge the food which collects between them, and which will speedily make vinegar having complete solvent power over them. A good dentifrice may well be used, as we have often said, but in the absence of exactly the right thing, a little soap or chalk will be found useful and harmless. We have often, of late, pointed out what we have found to be the best article prepared for use upon the teeth, and refer to the June number for our views in this regard. The great thing is cleanliness, unceasing energetic cleanliness. Without it no tooth is safe; with it, few teeth need be lost. These facts let parents command and teach and enforce by daily precept and by constant example.

---

RHEUMATIC FEVER. — Wonderful cures of this painful malady are reported to us on good authority, by the employment of the common Artichoke, the *Cynara* of the botanist. The leaves are used in an infussion, which is given copiously at any stage of the fever. They have to be gathered in their prime, just before the vegetable is ripe enough to eat. Ir left until the cone is fit for cooking purposes, the virtue of the leaves will have departed.

---

Sin is the fruitful parent of distempers, and ill lives occasion good physicians.

---

Prosperity seems to be scarcely safe, unless it be mixed with a little adversity.

---

Truth is the shortest and nearest way to happiness, carrying us thither in a straight line.

---

He who surpasses or subdues mankind must look down on the hate oi those below.

